# Children and Strong Drink

**Published:** 1920-08-21

**Authors:** 


**THE PUBLIC WELL-BEING—(*continued*).**

### Children and Strong Drink.

### A STRIKING EDUCATION SYLLABUS.

The syllabus of lessons for use in schools on the hygiene of food and drink, just issued by the Board of Education, certainly does not mince matters. Particularly on the subject of alcohol there is a downrightness of judgment which, while necessary perhaps for the purpose of creating a clear, unconfused impression on the mind of the child, might invite criticism from members of the medical profession, among whom there are still divided opinions as to the effects of alcohol in varying circumstances. In a prefatory note the Board point out that they have not hitherto recommended that temperance should be regarded as a separate and distinct subject of the curriculum, but it should form part of the general instruction given to children from time to time in the art of healthful living. The Board, however, consider that in view of the more important and comprehensive character of the syllabus, local education authorities should take such steps as are practicable to give it an appropriate place in the curriculum of the schools for which they are responsible, whether the instruction is given by members of the ordinary school staff or by specially appointed teachers of hygiene.

A justifiable warning is issued against the making of categorical statements or the drawing of inferences which are not supported by facts or fully substantiated by present scientific knowledge, and against the intrusion of "personal predilections and prejudices." One is bound to say that the syllabus, in spite of some possibly debatable assertions, is a good model in its clearness and restraint for the guidance of teachers, and gives them every opportunity of concentrating on the importance of self-discipline and avoiding the pitfalls of personal views, which, on the subject of intoxicating liquors, are apt to be held strongly on one side or the other. The syllabus deals in considerable detail with the nature and characteristics of alcohol; its effect on the brain and nervous system, on the body's strength and power to work, on the power of the body to resist disease, on the digestion of food, and on the body temperature; and a whole section is devoted to the misuse and abuse of alcohol, though we should imagine that this part of the syllabus—which examines mortality tables, the statistics of life insurance offices and friendly societies, the effect on national welfare and international competition, and so forth—is suitable only for the higher school classes, besides demanding considerable tact and ability on the part of the teacher in the exposition.

#### The "Drinking" Habit.

The following paragraph gives the key-note to the manner in which the subject is handled in the schedule:—

> Children and young people should not drink beer, wine, or spirits of any kind. Children do not, as a rule, like the taste of alcohol; in fact, wide experience proves that "alcohol is seldom any temptation to the young, but, nevertheless, the habit may be acquired and become a temptation later." [Quotation from the Right Hon. Charles Booth.] When in health the body does not need alcohol, whereas it is always possible that various evils may arise as a consequence of drinking beverages containing it. If alcohol is used

**THE PUBLIC WELL-BEING**—(*continued*).

regularly, "drinking" habits may not infrequently result, for a mere knowledge of the danger of alcohol is not a sufficient safeguard.

Here are two examples of the kind of illustrations employed as to the effect of alcohol on the body's strength and power to work:

> In the South African War it was observed by Sir Frederick Treves, who was with the column which relieved Ladysmith, that soldiers who drank much alcohol were the first to fall out on a long march, and were less fitted to overcome hardships and fatigue than those who either did not drink alcohol or took it in very moderate amount. . . .
>
> On the Great Northern Railway there was a celebrated gang of navvies who did more work in a day than any other gang on the line, and always left off work an hour or an hour and a-half earlier than any other men. Every man in this powerful gang was a teetotaler.

Useful illustrations, though the second is possibly open to the comment that the greater output of the teetotal gang may have been due quite as much to the will-power and force of character which made them band together as teetotalers as to their teetotalism.

